# Comparing perceptual segmentation in pulsed and unpulsed music with diverse event densities

**DOI:** 10.1007/s00426-026-02371-w

**Published:** 2026-09-11

**Authors:** Roger T. Dean, John R. Taylor

**Affiliations:** MARCS Institute for Brain, Behaviour and Development, Western Sydney University, Sydney, NSW Australia

## Abstract

Segmentation is a fundamental process in music perception, enabling listeners to identify meaningful boundaries within a continuous auditory stream. This process is crucial for perceiving rhythm and pulse, but more broadly, shapes both cognitive and emotional responses. In pulsed music, a clear, regular beat organises the rhythmic structure (and beat subdivisions as pulses are also normally regular in duration), whereas unpulsed music removes this overt timing reference (e.g., music with expressive rubato; non-isochronous or non-metrical music, traditional or new). While prior research has explored music segmentation, it has usually focused on pulse-driven responses. In this study, we examined how participants segmented diverse pulsed and unpulsed music and assessed the influence of event density (excerpts were grouped into sparse vs. dense groups), onset event patterns, metricality, acoustic features, and personal factors. Sixty-five non-musician participants (defined by non-enrolment in music programmes) listened to 26 musical excerpts and tapped to indicate perceived segmentation points. Bayesian autoregressive time-series analysis of inter-onset intervals revealed that in unpulsed music, segmentation is driven primarily by event density. The acoustic features: spectral centroid, spectral flux, and spectral complexity predicted segmentation boundaries, and RMS (acoustic intensity) showed autoregressive influence up to two lags. We also observed distinct differences in predictive factors between excerpts with sparse and dense event onsets. Personal variables (liking, familiarity, and perceived difficulty of tapping) did not account for additional variance in segmentation. Overall, our findings suggest that listeners use different segmentation strategies for music with sparse versus dense events, opting for a form of structural simplification when confronted with passages characterised by high event density. These results underscore the central role of musical structure in guiding event segmentation, and have implications in both musical and non-musical auditory contexts.

**Supplementary Information:**

The online version contains supplementary material available at https://doi.org/10.1007/s00426-026-02371-w.

## Introduction

Music listening involves segmentation, which is a term broadly described as the automatic and perceptual discretisation of sound events, and how they are organised for comprehension (Zacks & Swallow, 2007). The boundaries that delineate each discrete segment are a result of predictive hierarchical, recurrent, and cyclical perceptual information processing mechanisms moderated by attention and memory (Knösche et al., 2005; London, 2012; Zacks et al., 2007). Auditory events can be grouped together in a ‘phrase’, using pitch, loudness, and timbre (e.g. note- or sound-based music; Bregman, 1990; Cusack & Roberts, 2004; Krumhansl, 2000; Oh et al., 2022), or temporally grouped via either regularity, or the hierarchical organisation of sounds (e.g. rhythm-based music; Serman & Griffith, 2003; Tal et al., 2017; Zhang et al., 2016; Zuijen et al., 2004).

Most perceptual studies of temporal segmentation of music with respect to time, have focused on rhythm-driven responses in pulsed music. In this regard, they commonly concern the precision with which a listener can tap an auditioned rhythm, and in nearly all cases have used very simple rhythms, often repeating. For example, much of the previous research has commonly concerned tapping precision, notably sensorimotor synchronisation (Konvalinka et al., 2010; Repp, 2005; Repp & Su, 2013; Repp & Keller, 2008; van der Steen & Keller, 2013), accents (Jones & Pfordresher, 1997; Drake et al., 2000; Prince, 2014; Repp, 2005), pitch structure (Prince, 2014), phrasing (Knösche et al., 2005), and performance variations (Drake & Palmer, 1993), and the bulk of this work has mainly used very simple rhythmic stimuli. Some authors have investigated more challenging rhythms, whether constructed with isochronic (uniformly timed) ‘pulses’ (e.g. unfamiliar and complex rhythms; Milne et al., 2021) or with more variable inter-event durations. For clarity, we use the term *pulse* to indicate sonic events that form virtually isochronic series (where each successive pulse occurs with the same fixed temporal delay after its predecessor pulse). Once such series are established, occasional silent gaps in any pulse sequence also normally occupy multiples of the standard unit of time. ‘Beats’ are more emphasised cognitively, and are normally aggregates of pulses. Beats again remain virtually equally spaced in time (isochronic; Large & Palmer, 2002; London, 2012). In some rhythms, while pulses are close to isochronic, beats may contain varied numbers of pulses, usually in a repetitive organisation (e.g. repeating beats in the structure 3 + 3 + 2 pulses), and in some traditions there are well known small but consistent variations in pulse rate, in enunciating such rhythms (e.g. aksak; Holzapfel, 2015).

Our perceptible ‘beats’ (in our Excerpts 1, 2, and 26: see Table 1 below) are then usually comprised of aggregates of 2–3 pulses which are in turn of regular (isochronic) duration. While beats represent the cognised metrical structure of the music (such as potentially the 3 + 3 + 2 pattern just mentioned), many listeners still tend to emphasise pairs of pulses as their perceived beats, sometimes spread across what are metrically multiple bars: e.g. beats composed of isochronic pulses such as 3 + 2 | 3 + 2 can be perceived instead as 2 + 2 + 2 + 2 + 2 (Møller et al., 2021) or as more complex organisations (Milne et al., 2021). Beats may be accentuated in various ways, even though they are not always sounded but rather sustained virtually by the ongoing pattern. Note more broadly that rhythm is any controlled organisation of successive perceptible event durations, and as we emphasise in our choice of stimuli does not necessarily depend on the clearcut occurrence of (regular) pulses. Some of our dense stimuli (e.g. excerpts 3–8 in Table 1) really have few genuine regular timed pulses or beats, while having numerous energetic events; and when, conversely, events are sparse in time, we expect that participants will find it increasingly difficult to precisely identify any regular pulses that may occur. A general term for the time between two successive pulse or other event attacks is the IOI (inter-onset interval), and it is useful below in several contexts.

**Table 1:** List of stimuli and the YouTube URLs. Note that all audio and visual stimuli, as well as supplementary material can be found on the OSF data repository https://osf.io/ac4gu

Excerpt Number	Composer/ genre	Description	YouTube URL	Density/Pulsedness (PulseType)	MusicType	Duration (mm: ss)
1	Authors	simple meter	NA; Reference stimuli	Dense/pulsed	Western	01:00
2	Authors	a little syncopation	NA; Reference stimuli	Dense/pulsed	Western	01:00
3	Authors	Randomly timed drum, 1-strand	NA; Reference stimuli	Dense/unpulsed	Western	01:00
4	Authors	As for (3), 2-strand	NA; Reference stimuli	Dense/unpulsed	Western	01:00
5	Authors	As for (3), 3-strand	NA; Reference stimuli	Dense/unpulsed	Western	01:00
6	Authors	Random prime IOIs, 1-strand	NA; Reference stimuli	Dense/unpulsed	Western	01:00
7	Authors	As for (6), two strand	NA; Reference stimuli	Dense/unpulsed	Western	01:00
8	Authors	As for (6), three strand	NA; Reference stimuli	Dense/unpulsed	Western	01:00
9	Elliott Carter	Metrical modulation	https://www.youtube.com/watch?v=YKnE_Fj-agE	Dense/unpulsed	Western	01:00
10	LYSIS	Simultaneous multi pulse-rate jazz	https://www.youtube.com/watch?v=jksiAgZ-qas	Dense/pulsed	Western	01:15
11	Karlheinz Stockhausen	Sparse, restrained (part of Set Sail for the Sun)	https://www.youtube.com/watch?v=8dHpW7O1Olg	Sparse/unpulsed	Western	01:30
12	Evan Parker	Active sustained saxophone	https://www.youtube.com/watch?v=ByduB4Qpoug	Sparse/unpulsed	Western	01:30
13	Dave Burrell	Large group free jazz barrage	https://www.youtube.com/watch?v=Pc726YNjlM0	Sparse/unpulsed	Western	01:15
14	Iannis Xenakis	dense electroacoustic (Bohor)	https://www.youtube.com/watch?v=-wo8LeaUK94	Sparse/unpulsed	Western	01:20
15	Iannis Xenakis	dense orchestral (Metastasis)	https://www.youtube.com/watch?v=n2O8bMlEijg	Sparse/unpulsed	Western	01:14
16	A machine learning model, *Musicker.*	Chordal piano	https://www.australysis.com/private-view2021/ArtsScienceMilperraSessions.html (Video 3, at 36’10”)	Sparse/unpulsed	Western	01:00
17	Morton Feldman	Sparse chordal piano	https://www.youtube.com/watch?v=xx_gwPDgodU	Sparse/unpulsed	Western	01:30
18	Chinese traditional	Chinese traditional, sparse	https://www.youtube.com/watch?v=YHVt-xAaq-4&list=RDYHVt-xAaq-4&start_radio=1&t=33	Sparse/unpulsed	Non-Western	00:43
19	Riley Lee	Meditative shakuhachi	https://open.spotify.com/album/6FM6O1fbMalHJeUxXE5n3P	Sparse/unpulsed	Non-Western	01:58
20	Persian	Chant	https://www.discogs.com/release/1815444	Sparse/unpulsed	Non-Western	01:45
21	Louis Couperin	Unmeasured prelude for harpsichord	https://www.youtube.com/watch?v=K_G_k1WTdoc&t=14s	Sparse/unpulsed	Western	00:39
22	Traditional, King Shika	King Shika’s poem of praise	https://www.youtube.com/watch?v=5AXOpIsTGhU	Sparse/unpulsed	Non-Western	01:42
23	Javanese	Pathetan	https://www.discogs.com/composition/3f5353c7-bcd5-483d-b4f6-3482d134e3a5-Srimpi-Sangapati	Sparse/unpulsed	Non-Western	02:07
24	Indian	Rag puriya-kalyan	http://www.audiorec.co.uk/Inner-Voice-Budhaditya-Mukherjee-CD_0	Sparse/unpulsed	Non-Western	02:23
25	Turkish	Sufi Meditation	https://www.youtube.com/watch?v=-a_3ay8_76I	Sparse/unpulsed	Non-Western	01:44
26	Authors	Strongly 3/4 drums, multistrand; some complex syncopations, sometimes implying multiple pulse rates.		Dense/pulsed	Non-Western	01:00

In some previous work, Olsen et al. (2016) focussed on segmentation in circumstances where the clear note-based attacks that are usually central to rhythm generation and to perception of ‘pulse clarity’ were subordinated or even virtually absent. Furthermore, by contrasting note- and sound-based music, Landy (2011), suggested that the acoustic features that predicted segmentation vary considerably between different genres of music, notably Beethoven and Xenakis among others. In the present work, we wanted to take this consideration of segmentation a step further by comparing musical items with very different degrees of both event density and of pulse clarity, from simple two part keyboard repetitive isochronic patterns, through syncopated and then irregularly timed counterparts, to a multi-cultural group of pieces that do not espouse rhythmic drive or pulse, rather a more spacious temporal feeling, with continuous sounds, and sparse onset events often irregularly spaced in time. These included sparse keyboard music by Morton Feldman, dense electroacoustic continua in work by Iannis Xenakis, together with several examples of music from non-Western cultures that are commonly considered ‘*unpulsed’*: in our terms almost all music including these examples has occasional sparse onset events, but providing these events are not clearly regularly spaced in time, the music deserves this term *unpulsed* (and correspondingly is not beat based; Clayton, 1996; Widdess, 1994). 

We refer to the former group (Table 2 and 1–10, 26) of high density, sometimes pulse-driven pieces as ‘*Dense’ and either pulsed or unpulsed*: as we discuss later, the majority of the items in this group have IOIs randomised in various ways, and do not contain isochronic pulses or beats. The second, low density group, also lacking any pulse clarity in their events, is referenced as ‘*Sparse/Unpulsed*’, pointing to the fact that sequences of events may always occur where perceptually the inter-onset times are indistinguishable, but yet because of event sparsity, there may or may not be any generation of pulse clarity or perceived repetitive rhythmic drive. Indeed, our main purpose was not to define the degree to which listeners show such residual rhythmic perceptions, but rather to assess the time scales on which listeners still perceptually segment the music. This is partly because, as mentioned, there are a number of different types of music that are considered as having ‘free rhythm’ (being ‘ametric’), which is defined as music that lacks any periodic organisation (Clayton, 1996): either they are very sparsely pulsed, or a pulse or beat is entirely absent. Examples of these include religious music, such as: the *Ney Taksim* in Sufi meditation which is typically improvised (Güner, 2022); similar ‘free rhythm’ music from the Middle East, such as the Persian *avaz* (Clayton, 1996; Mirbagheri Fard & Reisi, 2023); the North Indian Ālāp (Widdess, 1994); Japanese shakahuchi and Chinese instrumental *qin* music (Clayton, 1996); Zulu poetry or *Izibongo* (Clayton, 1996; Pooley, 2016); and Javanese Pathetan (Brinner, 1989; Clayton, 1996), as well as unpulsed contemporary Western art music composers (cf. Dean & Bailes, 2010).

We addressed some specific questions of segmentation, where participants are asked (see below for detail) to tap when they perceive that a segment of auditioned time has passed. A segment can be taken as the period between two successive taps, or as in some analyses here, more conservatively as the period between successive time slices in which peak numbers of participants tapped. Consequently, we sought to answer the following questions:

1. Are there differences in the average segment duration according to degree of pulsedness, event density or sparse spaciousness? Note that participants may well sometimes tap regardless of the occurrence of a discrete/discernible acoustic event. This would be analogous to tapping of virtual beats in beat-based music.
2. Are segmentation patterns clearly related to detectable onset patterns? This embraces the question whether metrical or non-metrical pulsed stimuli necessarily induce metrical segmentation, and invites an assessment of the temporal (ir)regularity of tapping.
3. What acoustic or other features predict the occurrence of segmentation, represented here by taps?

## Methods

### Participants

This experiment was approved by our University’s Human Ethics Committee and participants provided informed written consent (approval number: H14126). Our sixty-five participants were first-year Bachelor of Psychology students. As none were enrolled in a tertiary music degree, we use the term “non-musician” throughout, which we consider as being independent of their measured musical sophistication. All participants were recruited via our university’s online participation system (SONA) and received course credit for participation. All participants met the minimum inclusion criteria for participation, namely self-reported normal hearing, and all participants conducted the test properly. Participants were asked to complete the Goldsmiths Musical Sophistication Index questionnaire pre-experiment (GMSI; Müllensiefen et al., 2014), to obtain information about their cultural background, and individual differences in musical sophistication. We administered a 31-item self-report portion of the GMSI (see Supplementary Material S1 https://osf.io/ac4gu), and results were consistent with participants’ non-enrolment in tertiary music programme, where the median lifetime instrument training was 6 months. For general musical sophistication (range 18–126), our participants spanned the full range of General Musical Sophistication (m = 81.65, sd = 14.35), which aligns closely with published population norms (m = 81.58, sd = 20.62; Müllensiefen et al., 2014). On the Musical Training subscale (range 7–49) our participants scored substantively lower (m = 18.37, sd = 9.37) than published population norms (m = 26.52, sd = 11.44; Müllensiefen et al., 2014), consistent with our characterisation of them as “non-musicians” by training and enrolment. The group was made up of 73.8% female, 21.5% male, and 4.6% preferred to not disclose their gender. Participant ages ranged from 17 to 48 (*m* = 22.6, s.d. = 6.43). Participant ethnicity/nationality percentages were: Australian (49.2%); Lebanese (6.1%); Filipino, Vietnamese, and Iraqi (4.6% each); Indian, Chinese, Greek, and Italian (3.1% each); and all others 1.5%.

## Materials

The experiment was developed using Max/MSP (Cycling’74, 2025), which was then deployed to a series of identical Apple MacBook Pro computers in our laboratory’s large group testing room. All workstations had the same headphones, mice, and operating system.

### Audio analyses

All audio files were processed using the *Essentia Standard Music Extractor* (Bogdanov et al., 2013) in Python, in which a selection of descriptors were extracted, and mean values calculated for each 500ms (2 Hz) window: centroid, energy, flatness, flux, spectral complexity, RMS, estimated fundamental frequency (F0_Pitch), and estimated fundamental frequency pitch confidence (F0_PitchConfidence). A Hann window was applied using Essentia’s default values - a frame size of 1024, and a hop size of 512 across the different lengths of the audio files with no zero padding.

### Audio event detection

To define the acoustic event boundaries of each excerpt, we used the Queen Mary University of London (QMUL) Vamp Plugin Note Onset Detector (Duxbury et al., 2003) in Sonic Visualiser (Cannam et al., 2010) to detect and extract onset points. Onset detection was undertaken again using a Hann window with size 1024, a hop size of 512, and a “Complex Domain” function using the mean of the source channels. Our objective was to determine whether or not each 500msec portion of an excerpt did or did not contain at least one onset (we did not aim to count the number of attacks within each 500msec chunk, and as discussed briefly below, Vamp sometimes missed several such attacks). All algorithmically identified onset containing chunks were visually and auditorily verified (and adjusted when necessary) by the authors, and the time annotation layer of the events by chunk was exported.

### Stimuli

Our auditory stimuli comprised both algorithmically generated stimuli, and diverse real-world created pieces. Our algorithmically generated excerpts (described further below) were designed as baseline rhythmic examples and used very similar sounds (e.g. the same drum types) to minimise the influence of acoustic features. The stimuli covered a range of different rhythmic and pulse variations and, as the trial sequence progressed, they became more rhythmically complex, giving participants ample opportunity to practice their segmentation with easier and more complex rhythms. For our real-world examples, we not only followed guidance from studies of world music (Ayari & McAdams, 2003; Lartillot & Ayari, 2011; Müllensiefen et al., 2014; Polak et al., 2016; Popescu et al., 2021), but we also undertook a search of accessible world music where we categorised our auditory stimuli as comprising either “dense” or “sparse” event sequences, and in terms of their pulse clarity. The “sparse” excerpts comprised predominantly continuous sounds, but with some sparse and irregular discrete events. Pieces from which the excerpts were chosen met three criteria: (1) that the genre of each excerpt was likely to be unfamiliar to participants (as our results confirmed); (2) that the stimulus set included diverse world music pieces, and (3) that the stimuli be roughly equally distributed between our “dense” or “sparse” categories. We included both pulsed and unpulsed, but with more emphasis on unpulsed, since this has lacked much prior study in relation to segmentation.

Potential excerpts were identified through a targeted search of publicly available recordings and archival collections, and were screened by the authors for our three criteria. Prior excerpts that exhibited clear metrical regularity, were of familiar western genres (e.g. Pop music) or contained prominent contemporary production effects, were excluded to avoid potential confounds. We also algorithmically constructed some examples of highly pulsed or high density unpulsed materials. Wav file versions of the stimuli were collected from a variety of online and fixed media sources, and imported into Cubase 11 Pro (Steinberg, 2025). For each stimulus, a short continuous (‘*sparse’* or ‘*dense’*, but not mixed) excerpt was selected that was representative of the prevailing event density and pulse characteristics of the piece, with a duration around a minute, and a maximum of 2’23” to ensure our participants could undertake the experiment within a maximum of 1 hour.

We also simply categorised our auditory stimulus (*MusicType)* as being either “Western” or “Non-Western”, guided by the origins of either the musical genre/style, lyrics, or instrumentation (e.g. excerpt number 20, Persian chant, is considered “Non-Western”, whereas excerpt number 14, Xenakis’ “Bohor” electroacoustic piece is considered “Western”). A list of the auditory stimuli is shown in Table 1, together with a brief description of the piece, a URL to an online example (if available), our *Density/Pulsedness* (*PulseType*) category, our *MusicType* category, and Duration (minutes and seconds). 

There were also three visual stimuli, intended as a pilot test, which are included in the https://osf.io/ac4gu.online supplementary material. These comprised a series of identical vertical lines organised in a horizontal sequence across the page (from 8 to 15 lines, regular or irregular in spacing). Our original aim was to test whether our participants could translate horizontal distances into temporal gaps, and tap regular or irregular segments of time that corresponded to the distances. However, our instructions were inadequate and misunderstood. Most participants tapped about twice per line, and many participants tapped far more segments than there were lines (max was 148!). Previously, Grahn et al. (2011) found that implied beat tempo is far less detectable in visual sequences than in auditory sequences, yet visual statistical learning can be improved when preceded by auditory sequences of the same temporal structure. Other work on visual rhythm has used a variety of stimuli, ranging from simple flashing dots (Patel et al., 2005), or black squares (McAuley & Henry, 2010), to more sophisticated rhythmic representations of objects moving to different positions (Schubotz et al., 2000). A different design from ours is required to pursue our visual-rhythm questions meaningfully, and we do not present any results from these responses.

### Procedure

Prior to undertaking the experiment, participants were briefed about the aims and requirements of the study and screened to ensure they met the inclusion criteria. Participants provided written informed consent before taking part. Participants completed an online version of the Goldsmith’s Musical Sophistication Index (GMSI; Müllensiefen et al., 2015) prior to completing the listening experiments, to confirm their level of musical sophistication. On-screen instructions were given before the participant began listening to the first excerpt, as follows:

*“Rhythm is the perceptual segmentation of time as a consequence of the organisation of (musical) sounds in time. These sounds may be regularly or irregularly spaced in time, frequent or sparse, continuous or discontinuous; and a previous sonic pattern may be suspended for a while or replaced. The same is true of rhythm. Each listener perceives rhythm their own way: there is no right and wrong. Task: first, you will hear a 30 s audio clip for familiarisation. Then, you will hear the same audio clip for approximately 60 s. When you hear it the second time, tap on the spacebar each time you feel a segmentation point. There may not always be an event ending there, as a rhythmic pattern may seem to continue through a rest or silence. Don’t tap while you do not envisage a segmentation point.”*

Each tap time (measured from the start of the audio excerpt) was captured and saved to a .csv file. For each trial, participants were allowed to hear the first 30 seconds of each stimulus for familiarisation, followed by the full excerpt of the stimulus in it’s entirely. We captured all tap data, including those taps made during the initial 30 second audition of each excerpt, and the subsequent full excerpt. The tap data for the 30 second rehearsals of each excerpt were not included in the final dataset. The small number of participants who did not tap during a given excerpt were not treated as non-compliant. Instead, taking account of researcher observations we considered the absence of tapping in a few trials to be a valid response indicating that no salient segmentation boundary was perceived within that excerpt. For example, from an auditory scene analysis standpoint, sustained non-tapping might reflect continued stream coherence rather than participants misunderstanding of, or failure to complete the task. Consequently, responses with no taps were retained in our analyses (including in aggregate statistics): omitting them could bias analysis towards event-driven segmentation strategies. (Actually, analyses conducted after such no-tap response series were excluded showed no substantive changes in predictors and interpretation.) Participants initially completed excerpt numbers 1–4 in order, then the three visual “rhythms”; followed by excerpts 5–26: this order was fixed, so that they had experienced the items with pulse clarity in the early stages. After the listening to each full excerpt, participants were asked to rate how much they *Liked* each excerpt, how *Familiar* they were with the music, and how *Difficult* the tapping task was for each excerpt. Ratings for each of these were on a Likert scale 1–7 (1 = not at all liked/familiar/difficult; 7 = very liked/familiar/difficult; see also below the section ‘Participant Features and Responses’). After completing the experiment, participants were debriefed.

### Data analyses

Our data are in three broad categories: segmentation/tapping information, stimulus related (e.g. acoustic features), and participant features (e.g. cultural background, musical preference and sophistication). The segmentation/tapping data comprise: *Index* which refers to counts of ‘time’ in 500ms intervals across an excerpt; the variable *MTP/slice* is the mean tap per slice, and the variable *CVTP/slice* is the corresponding coefficient of variation in that number (s.d./mean). The variable *nLPK* refers to the number of large peaks of coincident taps within a segment, and *mIOILPK* is the mean inter-onset interval (seconds) of these ‘large’ peaks.

Our stimulus data included: *PulseType*: whether or not the musical stimuli are pulsed.

Note again that the dense group includes several stimuli that comprise dense events, but technically several of these are not regularly placed in time, hence not strictly pulses, but may be perceived as such. *MusicType* denotes whether the musical stimuli are of “Western” or “Non-Western” origin *Event_Prop*, the proportion of time slices that contain an acoustic event. *Event_Onset* is the edited QMUL Vamp-detected binomial segmentation marker that indicates whether an acoustic onset was detected in each time slice (1 = onset, 0 = no onset), as discussed above. While we observed that many Vamp-detected events arguably comprised a perceptible group of events, we only made edits to the rare case that Vamp missed all such events within a time slice. The *Inter_Onset_Interval* or *IOI* refers to the interval between each *Event_Onset*, measured in either seconds (s) or milliseconds (ms). The variable *tAfterOnset* is the time slice count after the last *Event_Onset*. For example, in the time slice immediately after a time slice where *Event_Onset* = 1, *tAfterOnset* = 1, for the next time slice, *tAfterOnset* = 2 and so on, until another segmentation or a new Event_Onset occurs. *Time* refers to the current time index of each event, measured in 500ms (2 Hz) increments, starting at zero to n, the total number of time slices in the excerpt.

We used several Essentia acoustic measures to describe the stimuli at a 2 Hz sampling rate, each standardised to a mean = 0, and s.d. = 1. Although the acoustic features were analysed at a 2 Hz (500ms) temporal resolution, each time slice value represents the mean of multiple overlapping STFT frames computed within each 500ms window, using a Hann window (frame size = 1024 samples; hop size = 512 samples) as described in the audio analyses section above. Accordingly, acoustic estimates at each time index are based on several short-time analyses spanning the full 500ms interval, rather than on a single instantaneous measurement of 500ms in length. As we describe later, these acoustic feature predictors are then either used as excerpt-level ‘grand mean’ values (denoted by a preceding ‘m’; e.g. *mCentroid*, *mEnergy* etc.); or for the time series analyses, as lagged values from time slices, for example: CentroidL1 (Lag 1) is the predictor *Centroid* from the previous time slice, CentroidL2 from two time slices ago, while CentroidL0 would be the current time slice etc. *Centroid* or ‘spectral centroid’ is the frequency centre of gravity of the signal’s spectrum and is typically used as a measure of brightness (e.g. a higher *centroid* measure indicates brighter, higher frequency). *Flatness* or ‘spectral flatness’ is a measure of how flat a spectrum is, calculated as the ratio of the geometric mean to the arithmetic mean of the power spectrum, where higher values indicate a flatter (noisier) spectrum, and lower values indicate a more harmonic signal. Spectral *Flux* is a measure of how quickly the power spectrum changes over time, typically calculated as the squared difference between the magnitudes of successive spectral frames, indicating the rate of spectral variation. *Flux* here uses the L2-norm (Euclidean distance) as described by Tzanetakis and Cook (1999), and higher measures of spectral *flux* typically correspond to increased rhythmic complexity. *F0_Pitch* is the fundamental frequency, which is the lowest frequency of a periodic waveform, representing the pitch of a sound. *F0_Pitch* was calculated using Essentia’s *PitchYinFFT* (Brossier, 2006) and is represented in Hz. Furthermore, Essentia’s *PitchYinFFT* also provides the variable *F0_PitchConfidence*, which is a measure of the confidence with which the pitch was detected (0 = not confident, 1 = very confident). *RMS_Loudness* (Root Mean Square) is a statistical measure of the magnitude of a varying signal and provides a normalised (averaged) measure of a signal’s energy, which more closely resembles perceptual loudness. *Spectral_Complexity* is the number of peaks in the input spectrum, with more peaks indicating more frequency components, and increased spectral complexity (Laurier et al., 2010).

### Participant features and responses

Before the experiment we obtained participants’ GMSI data. After each trial, we asked participants to rate their *Liking* of and *Familiarity* with the excerpt they had just heard, as well as the *Difficulty* they had tapping the segmentation points for it (1 = not liked/familiar/difficult, 4 = somewhat liked/familiar/difficult, 7 = very liked/familiar/difficult).

### Analytical design

‘Grand aggregate’ time series were constructed for each piece that represented at 2 Hz sampling whether or not a time slice contained a computationally detectable event, and how many participants tapped within the slice. Note that this is similar to the common approach of constructing a grand average response time series, by simply averaging, point by point, all the individual (fully aligned) time series. Corresponding acoustic information was also assembled. Such basic descriptive statistical data provide considerable insight into the contrasting responses here to different pulse densities. Models were also conducted on the multiple participants’ responses to each piece, in a multilevel mixed effects analysis in which each response retained its integrity (no averaging or summing). In these models, we assessed possible influences of acoustic and other features using multilevel Bayesian autoregressive time series analysis with the R package *brms* (Bürkner, 2019) which provides a high-level interface to the Bayesian MCMC sampler s*tan* (Carpenter et al., 2017). The models were so-called maximal models, in which group (random) effects on participant and excerpt were included. These effects supported the indications of inter-individual and inter-excerpt variations in influential features.

Time series models were computed as Poisson (count) series and were also checked for overdispersion using leave-one-out (loo) cross validation in *brms*, and were appropriate remodelled using family = negbinomial. A standard model was adopted as a shared assessment, involving autoregression of the response (when detected initially, or if found necessary because residuals of the model otherwise remained autocorrelated), together with acoustic features, and the acoustic event marker. Vamp-detected binomial markers (after the minor editing by expert listeners) indicated whether an acoustic onset was detected in each time slice (as discussed above), and where appropriate, models used to assess time series dependencies on the occurrence of these binary markers were computed using the brms family=bernoulli. When such onset events are present in virtually every time slice (as in Piece 1; see Table 2), they provide very limited information, and so it is unlikely that they will be strong predictors of the incidence of taps in comparison with acoustic features such as RMS intensity changes. But when events are somewhat sparse, they might be expected to function like the events of interrupted time series (like the establishment of a new treatment for novel disease, or the initiation of a new road speed limit). That is, potentially they may create two main effects: an immediate change in the level of the response variable, and a progressive change thereafter (in this case, hypothesised and confirmed to be downwards). Thus, it is normal to include in the model a predictor that represents time since the last event and based on preliminary modelling, we did so.

A reviewer pointed out that an extreme interpretation of our data, given the stimuli were presented in a fixed order, could be that the main differences between the dense and sparse stimuli were due to their position in the sequence. However, note that among the dense stimuli, one was placed at item 26, whereas the others occupied items 1–10: yet the results for 26 were very closely related to those for 10, as expected by the features of the items. This makes strong order dependence unlikely. Furthermore, for control analyses of the time series (extending those in Tables 7 and 8) we also constructed a continuous time index by sequentially summing the individual time indices of the items, and repeated the basic analysis using this index with both the two separate (dense, sparse) datasets, and with a combined sequential dataset containing both. Whereas time progression within individual trials was a significant influence as we show below, this sequential cross-item time measure was not (not shown). Thus, we consider the results and our interpretations unlikely to be substantially affected by stimulus order.

We computed a variety of different models, using an additive approach to predictor inclusion: some models were useful and reported, others were not, some failing to converge, and are only reported if necessary. We used small uninformative priors (those which provide little specific information beyond supporting the idea that coefficients should be small, which is highly appropriate with standardised or binomial data). We also confirmed empirically that a range of priors provided only slight quantitative differences in measured coefficients (i.e. in the posterior results). In general, we used the brms default priors. For one-sided Hypothesis tests, a 95% posterior probability that $\:\beta\:\:>0$ yields an evidence ratio of $\:\frac{0.95}{1-0.95}\:=19$, which we consider ‘strong’ evidence. For two-sided Hypothesis tests, if the posterior probability that $\:\beta\:\ne\:0\:$exceeds 0.975, the evidence ratio is $\:\frac{0.975}{1-0.975}\:=39$, equally taken to indicate ‘strong’ evidence.

## Results

### Comparison of the grand aggregate time series for tapping segmentation of two contrasting pieces

**Fig. 1:**
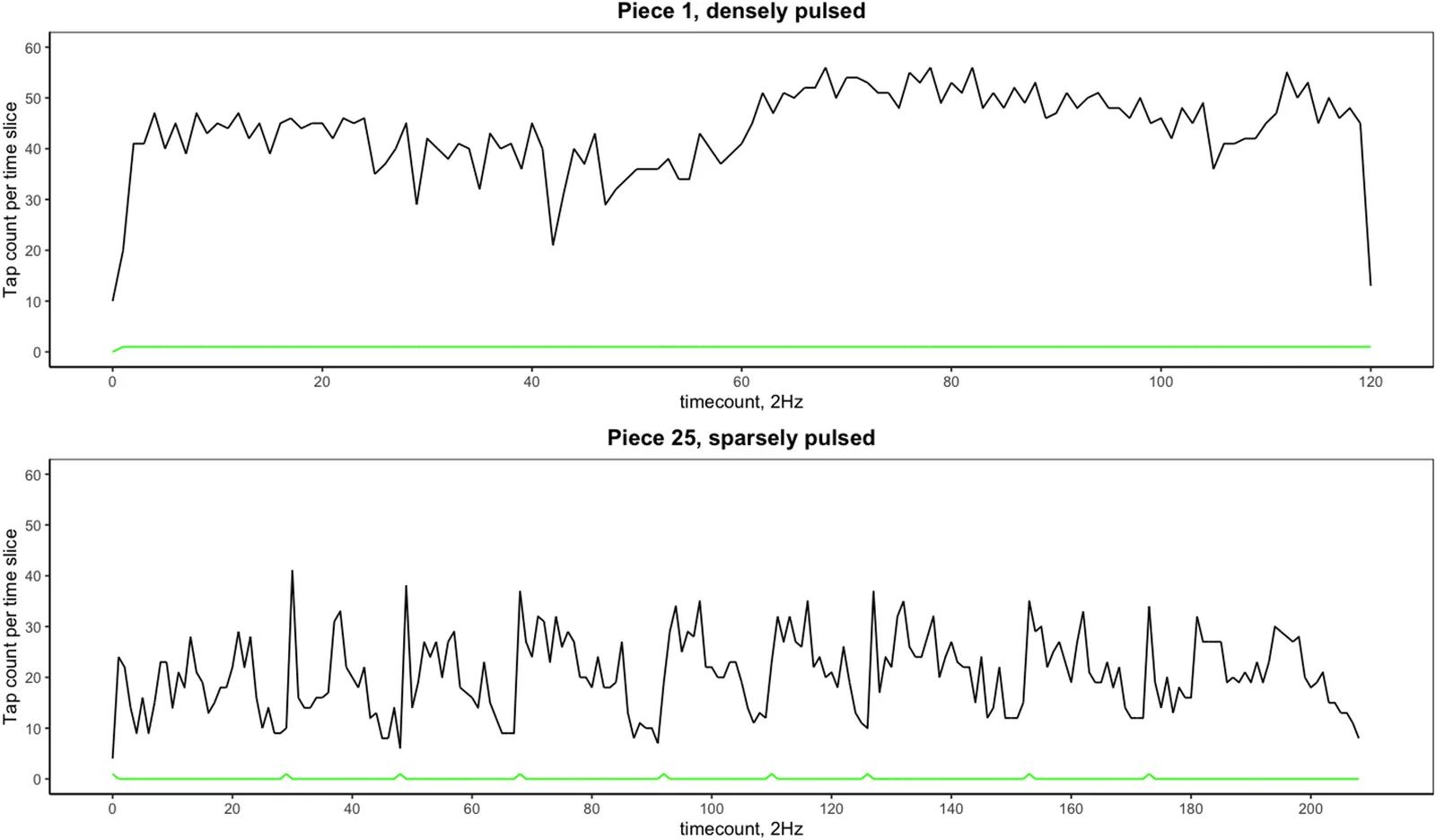
Aggregated tapping responses to two contrasting pieces. Tap counts per 500ms time slice (black) are compared with the incidence of computationally identified acoustic events (green), which occur in every slice of Piece 1 (metrical) except the first. The sparse acoustic events in Piece 25 (Sufi meditation) contrast extremely, as does the pattern of tapping

Figure 1 compares the aggregate time series results for two cases, one being the simplest densely pulsed rhythmic and metrical item (1), the other an arbitrarily selected member of the sparsely pulsed group (excerpt 25). Piece 1 is a simple algorithmic piece with a very regular pulse rate throughout. Most acoustic events are explicitly spaced at 500msec (120 per minute), and judging by the response frequencies, perceived mainly as 4/4 crotchets (quarter notes i.e. also perceived as beats) as shown by the recurrent pattern of high/low/medium/lowest tap counts. The large drop in count at index 43 initiates an acoustic gap, when participants typically produced a segmentation tap at or immediately preceding the onset of a gap, followed by a marked reduction in tapping during the gap itself. It appears that segmentation in this beat-oriented piece was indicated by discrete taps marking perceived boundaries, with taps subsequently withheld until the next perceived event. The progressive increase in tap counts at c. timecount 63 onwards and c. 105 onwards each coincide with increasing numbers of sounded 16th notes (semiquavers) in relation to the 120 per minute beat and taps. Piece 25, a Sufi meditation, contains drone and a melodic wind instrument, and only 9 time slices contain detected acoustic events. These are each the beginning of a continuous melodic phrase. Clearly, listeners perceived these points as segmenting, and after each such event tap counts generally declined until the next. In those terms, some arguable acoustic events perhaps go computationally undetected (e.g. around time count 195). Generally, tapping is far more sparse (less than half the average rate) than in the densely pulsed piece, again consistent with listeners generally perceiving much longer segments.

### Segment durations and average tapping rates in the grand aggregate data

The implications of Fig. 1 follow through more generally when we consider aggregate time series of individual stimuli. Tables 2 and 3 summarise such statistics obtained from the aggregate time series for each stimulus. Table 2 describes the group of 11 ‘control’ dense pieces, that have conventional note-based instruments and strong rhythmic elements, from metrical to non-metrical (hence pulsed to unpulsed). It is important to note, as we stressed in the introduction, that here 'densely' means 'densely laden with events', and does not necessarily imply the presence of meter or a regular beat, as particularly with stimuli 3–5. Table 3 describes the 15 diverse sparse pieces chosen because of their sparseness, or spaciousness, belonging to styles that are often described as ‘*unpulsed’*.

Among the salient features of the data of Table 2 are the relatively high event and tap density for most pieces, especially where regular pulse is clear-cut. When computationally randomised timings are introduced (group 3–5, group 6–8), the proportion of time slices containing an event is reduced, but raised again progressively as multiple strands are introduced and event density rises: correspondingly taps per/slice start low, and rise. The highest MTP/slice occurs with a jazz piece (10) with frequent acoustic events, and is unperturbed by the introduction of multiple pulse rates after about 20 seconds, showing the lowest CVTP/slice in the set. Conversely, Elliot Carter’s highly systematic metrical modulation produces more event sparsity, and a low MTP/slice, roughly equivalent to the one strand randomised drums of piece 3. Item 26 produces very strong tapping even though relatively few acoustic events are registered. Somewhat as with items 1–3, where only beats are detected (and not subdivisions) the onset detector acts in 26 like a downbeat (opening beat of the 3/4 bars) detector, and disregards several other events. Tapping recognises these peaks, but also events between them. 8 of these pieces have 4 or less ‘large’ tapping peaks (characterised by an abrupt proportionally large increase in the number of tappers: see Fig. 1 legend), which are thus uninformative. The remainder are among the pieces with lower Event_Prop, and constitute three of the four lowest MTP/slice, indicating tapping sparsity compared with other pieces, and corresponding to their randomised rhythm patterns. Overall, one can observe that tapping is heavily dependent on rhythmic structure but represents it in a highly simplified way: sometimes this coincides with a compositional view of metrical salience, but not always. The quite low CVTP/slice values (mean 0.23, maximum 0.48) suggest general agreement among participants. Complex or irregular rhythms clearly disrupt listeners’ perceptions, extending the disparities between them.

**Table 2:** Summary Statistics for pieces with densely pulsed elements

Piece	Length	Event_Prop	MTP/ slice	CVTP/ slice	mIOILPK	nLPK	Description
1	121	0.99	43.70	0.18	Inf	0	Simple meter
2	121	0.99	44.30	0.15	Inf	0	A little syncopation
3	121	0.62	30.40	0.31	3.28	16	Randomly timed drum; 1-strand
4	121	0.89	37.30	0.17	60.00	1	As for 3, 2-strand
5	121	0.98	38.40	0.15	29.75	2	As for 3, 3-strand
6	121	0.42	25.60	0.48	2.52	20	Random prime number ratio temporal intervals, 1-strand
7	121	0.70	32.60	0.25	5.00	11	As for 6, 2-strand
8	121	0.85	36.90	0.20	14.62	4	As for 6, 3-strand
9	121	0.58	30.90	0.28	19.67	3	Elliot Carter; metrical modulation
10	150	0.99	49.20	0.07	Inf	0	LYSIS; multi-pulse-rate jazz
26	121	0.32	45.40	0.19	Inf	0	Strongly 3/4 drums, with complex cross-rhythms; multistrand

Length is in timeslice counts (2 Hz). Event_Prop is the proportion of time slices that contain an acoustic event. MTP/slice is the mean number of individuals tapping per slice, and CVTP/slice the corresponding coefficient of variation in that number (s.d./mean). mIOILPK is the mean inter-onset interval (seconds) of ‘large’ peaks (of which there are nLPK) in the number of people tapping, as illustrated in Fig. 1 for Piece 25. A large peak is defined as one which has an increase of > = 50% of the mean taps/slice for that piece from the immediately preceding time slice, and which is immediately followed by a tap count decrease (of any magnitude). As seen in Table 3, this is particularly relevant for the spacious pieces with relatively low MTP/slice. The pieces are described fully in Methods

Table 3 is in high contrast to Table 2, with all but two stimuli showing much lower proportions of time slices containing a detected event; and with generally lower MTP/slice (range 10–44, vs. 25–49 in Table 2). Most revealing is the more frequent occurrence of ‘large’ tapping peaks (nLPK), particularly in the low MTP/slice cases, and their continued long duration (mostly from 4 to 80 seconds). Clearly, this was not due to rhythmic confusion, as one could view the occurrences in the randomised but still strongly pulsed items in Table 2. Rather, it was connected with a sparsity of clearly distinct ‘new’ acoustic events. As mentioned already, while Vamp quite often missed perceptible events, it only very rarely entirely missed a group of events within a time slice, and these cases were manually edited when necessary. Thus, missed Vamp onset detections in certain stimuli do not compromise the validity of our dense–sparse classification.

**Table 3:** Summary Statistics for sparsely pulsed pieces, highly spacious or continuous

Piece	Length	Event_Prop	MTP/ slice	CVTP/ slice	mIOILPK	nLPK	Description
11	181	0.15	24.26	0.24	22.12	4	Stockhausen; sparse, restrained
12	181	0.20	44.00	0.10	Inf	0	E. Parker; active sustained sax
13	151	0.05	40.11	0.10	Inf	0	D. Burrell; large group barrage
14	161	0.08	21.87	0.14	80.00	1	Iannis Xenakis; dense electroacoustic
15	298	0.15	22.68	0.38	6.27	22	Iannis Xenakis; dense orchestral
16	121	0.50	27.53	0.33	4.54	12	Musicker; chordal piano
17	181	0.08	10.29	0.84	4.82	17	Morton Feldman; sparse chordal piano
18	86	0.36	22.37	0.53	3.80	10	Chinese traditional.; sparse
19	238	0.03	22.70	0.35	9.42	12	Riley Lee; meditative shakuhachi
20	210	0.38	37.77	0.25	25.75	4	Persian chant
21	79	0.63	31.63	0.21	39.00	1	Couperin; unmeasured prelude
22	205	0.42	39.31	0.17	20.00	5	King Shika’s praise poem
23	256	0.04	31.53	0.22	Inf	0	Javanese Pathetan
24	287	0.13	24.33	0.55	2.55	47	Rag puriya-kalyan
25	209	0.04	20.49	0.36	12.56	8	Sufi meditation

Xenakis’ item 14 could be construed as having multiple (electro-)acoustic events per second, but the majority of participants did not segment the piece with such event-level granularity: instead, they segmented at larger-scale perceptual units. It is important to note that participants were instructed to mark perceived segmentation boundaries and were not constrained to a specific level of musical structure. This is because we did not wish to assume a priori that segmentation boundaries corresponded to individual low-level acoustic events rather than phrase-level segments, since this perceptual ambiguity is a central area of interest in this study.

We deduce from the data on items 12–13 that tapping, and hence representation of segmentation, can be encouraged by high activity and acoustic intensity (see modelling to follow), while the other items indicate that spacious, especially meditative musics, are generally perceived as comprising longer segments. This reflects participants’ tendency to group extended regions of relatively stable sound into single longer units (similar to a perceptual stream in auditory scene analysis), rather than segmenting based on individual acoustic events. While this is an indirect deduction from the aggregate data analyses and the comparison of pieces 1 and 25 in Fig. 1, it reflects more broadly the differences in listeners’ sound organisation and segmentation strategies between excerpts with varying event density. This inference should not be interpreted as a correlation between acoustic event count (which we did not try to measure) and segment length (as noted above we did not adjust Vamp onset detections to assess the multiplicity of onsets within each 500ms analysis window, rather solely to ensure that when there was one or more events the window was identified as event-containing). The degree of agreement between participants (as reflected in the higher mean CVTP/slice of 0.32) was lower than in the densely pulsed pieces. In the next section, we make direct assessments of the inter-onset intervals (IOIs) of individual tapping across all the pieces.

### Direct assessment of tapping IOIs for individual pieces across participants

For each piece we first assessed the mean tapping IOI for each participant. We next present statistics across participants (mean, s.d. and maximum of the individual means).

The very varied responses across participants in Table 4 confirmed that the denser, more pulsed pieces (1–10, 26), tended to have quite low mean ITIs from about 1 to 3.5 s. In contrast, 7 of the spacious pieces 11–25 had means greater than 3.5 seconds, with a maximum in item 14 of about 13.5. The minimum value in this group (3.84, item 24) corresponded to about 1.9 seconds. Importantly, these differences suggest that dense and spacious works elicit qualitatively different segmentation strategies in listeners, consistent with differences in perceptual organisation. In this sense, our results provide empirical support for Q1 by constraining how musical density relates to segmentation behaviour. With regards to our Question 2, that the frequency of computationally identified onsets (supported by expert listener consideration) relates to the frequency of tapping, we found only partial support. Consequently, we use mechanistic models in the next section to examine the influence of acoustic features on segmentation (Q2 and Q3).

**Table 4:** Inter-tap intervals (units of timeslice counts) from 65 individuals, for each of the 26 pieces

Piece	mean	s.d.	max	Description
1	2.42	3.41	20.17	Simple (2/4) meter
2	2.16	2.37	13.44	A little syncopation
3	7.12	21.22	121	Randomly timed drum; 1 strand
4	6.07	20.75	121	As for piece 3; 2 strand
5	6.24	20.91	121	As for piece 3; 3 strand
6	6.11	16.48	121	Random primes-timed IOI; 1 strand
7	4.53	14.84	121	As for piece 6; 2 strand
8	4.23	14.9	121	As for piece 6; 3 strand
9	5.91	16.42	121	Metrical modulation
10	2.46	3.84	21.43	Multi pulse rate jazz
26	3.28	6.74	40.33	Strongly 3/4 drums; multistrand
11	9.32	23.14	181	Sparse, sustained, restrained
12	2.90	3.48	15.08	Active sustained saxophone
13	10.18	31.9	151	Large group free jazz barrage
14	27.02	52.11	161	Dense electroacoustic
15	7.35	18.44	149	Dense orchestral
16	4.15	7.65	60.5	Chordal piano
17	15.09	24.5	181	Sparse chordal piano
18	4.31	3.74	17.2	Chinese traditional; sparse
19	15.81	49.57	238	Meditative Shakuhachi
20	3.86	7.11	42	Persian chant
21	4.54	9.90	79	Unmeasured Prelude for harpsichord
22	8.61	35.32	205	King Shika’s poem of praise
23	4.12	4.63	23.27	Javanese Pathetan
24	3.84	3.13	17.94	Indian Rag puriya-kalyan
25	17.34	44.92	209	Turkish Sufi meditation

For each piece 1:26, an average, s.d., and maximum inter-tap interval (ITI) was determined for each participant. The units are 0.5 seconds, the 2 Hz analytical time slice. From that, the overall average of 65 values for mean and s.d., and the maximum are presented. Some of the Maximum (max) values indicate that at least one participant did not tap at all during the piece in question: this occurred in the more rhythmically complex/unfamiliar pieces 3–9, as well as in 9 of the ‘spacious’ pieces. Responses were very variable across participants, as indicated by the fact that in all but 2 cases, the coefficient of variation implied (s.d./mean) is much greater than 1. The patterns of mean ITI predicted by the preceding discussion are clearly confirmed. The upper part of the table are considered the ‘dense’ group (items 1-26) and the lower part of the table (items 11-25) are considered the‘sparse’ group of stimuli. A superficial comparison of the two groups overall can be made by noting the respective means of the tabulated mean values: it is 2.26 seconds for the upper dense and 4.51 for the lower sparse groupthe 2 Hz analytical time slice. From that, the

#### Bayesian time series analysis of Poisson event frequencies in grand-aggregate time series of tapping of individual pieces

As one approach to identifying mechanistic impacts on segmentation responses of the Vamp-detected onset events and the acoustic features, we first describe models of the grand aggregate time series of tapping piece by piece, the data used extensively above. To analyse the major contrasts described already, we particularly consider pieces 1, 3, and 6 among the rhythmic set; and 11, 14 and 25 among the sparsely pulsed set.

A standard model was adopted as a shared assessment, involving autoregression of the response (when detected initially, or if found necessary because residuals of the model otherwise remained autocorrelated), together with acoustic features, and the acoustic event marker. This Vamp-detected binomial marker indicates whether or not an acoustic onset was detected in each time slice, as discussed above. When such events are present in virtually every time slice (as in Piece 1; see Table 2), it is unlikely that they will be strong predictors of the incidence of taps in comparison with acoustic features (such as RMS intensity changes). But when events are somewhat sparse, as described already they might be expected to function like the events of interrupted time series. That is, there are potentially two main effects: a fairly immediate change in the level of the response variable, and a progressive change thereafter. Thus, we include the predictor that represents time since the last event (tAfterOnset) in the model. Acoustic features and the ‘time’ variable were standardised, and minimally informative priors chosen. Two lags of the RMS (acoustic intensity measure) were included.

Table 5 shows that Event_Onset1 and tAfterOnset have zero coefficients. Instead, key drivers of the segmentation response seem to be RMS, spectral centroid, and spectral flatness. Hypothesis tests in Table 6 confirms this.

**Table 5:** Standard Model of the grand aggregate tap count time series for Piece 1

Family: Poisson Links: mu = log Formula: tap_count ~ Age*Event_Onsets + tAfterOnset + RMS + RMSL1 + RMSL2 + Centroid + Flatness + Flux + Spectral_Complexity + Index + Liking + Familiarity + Difficulty + AR(*p* = 4) + (1 | PID) + (1 | Track_ID)							
Correlation Structures:							
	Estimate	Est. Error	l-95% CI	u-95% CI	Rhat	Bulk_ESS	Tail_ESS
AR[1]	0.01	0.55	-0.94	0.94	1.00	33,442	11,365
AR[2]	0.02	0.55	-0.94	0.94	1.00	34,576	11,240
Std. Error	0.01	0.01	0.00	0.03	1.00	14,306	10,951
Population-Level Effects:							
	Estimate	Est. Error	l-95% CI	u-95% CI	Rhat	Bulk_ESS	Tail_ESS
Intercept	3.77	0.50	2.80	4.74	1.00	46,607	13,022
Event_Onset1	0.00	0.50	-0.97	0.97	1.00	46,641	12,827
tAfterOnset	0.00	0.50	-0.98	0.99	1.00	48,425	12,804
RMS	-0.08	0.04	-0.16	-0.00	1.00	17,479	14,987
RMSL1	0.08	0.02	0.05	0.11	1.00	32,256	14,745
RMSL2	0.04	0.02	0.00	0.07	1.00	38,815	13,907
Centroid	0.10	0.08	-0.06	0.26	1.00	19,634	13,964
Flatness	-0.26	0.09	-0.43	-0.08	1.00	18,640	13,918
Flux	0.01	0.02	-0.02	0.04	1.00	30,201	14,598
Spectral_Complexity	0.06	0.03	0.00	0.13	1.00	20,434	15,505
*Time*	0.04	0.02	-0.01	0.08	1.00	18,658	15,365

Table 5 legend. Note that AR=autoregression, and L*n* denotes a lag number. Note also that the model Bayesian R^2^ = 0.766

**Table 6:** Hypothesis testing for standard model for Piece 1

Hypothesis	Estimate	Est. Error	CI. Lower	CI. Upper	Evid. Ratio	Post. Prob	Star
Event_Onset > 0	0.00	0.50	-0.82	0.81	1.00	0.50	
tAfterOnset < 0	0.00	0.50	-0.83	0.82	0.99	0.50	
RMS < 0	-0.08	0.04	-0.14	-0.02	44.57	0.98	*
RMSL1 > 0	0.08	0.02	0.05	0.11	Inf	1.00	*
RMSL2 > 0	0.04	0.02	0.01	0.07	48.72	0.98	*
Centroid > 0	0.10	0.08	-0.03	0.23	8.44	0.89	
Flatness < 0	-0.26	0.09	-0.41	-0.11	427.57	1.00	*
Flux > 0	0.01	0.02	-0.02	0.03	0.51	0.34	
Spectral_Complexity > 0	0.06	0.03	0.01	0.12	51.02	0.98	*
Time > 0	0.04	0.02	0.00	0.08	16.60	0.94	

There was strong evidence (evidence ratio > 19 for a unidirectional hypothesis, and > 39 for bidirectional) for effects of RMS, spectral flatness (negative coefficient), and spectral complexity. There was no evidence for effects of Event_Onset, immediate or delayed, as expected. While this model formula is often redundant (i.e. contains some predictors with no or little effect), it properly tests the questions of interest, and provided it gives a model comparable with the best we could obtain, we present it. The inclusion of autoregressive terms, on the other hand, is decided for each model as we have mentioned already. For the later models in this section of analyses, we summarise by solely describing the predictors with strongly evidenced coefficients within the standard model, and we specify the AR terms required, and the Bayesian R^2^ degree of fit.

For piece 3, with randomised-time single strand drumming, the standard model (with AR1) gave a Bayesian R^2^ of 0.553, which is reasonable. Now, given the sparser (randomised) events, tAfterOnset became highly evidenced as a negative influence (estimate=-0.39, evidence ratio = 1499.00,), RMSL1 was highly evidenced (estimate = 0.10, evidence ratio = 999), as was *Time* (estimate = 0.05, evidence ratio = 81.57). This is clearly consistent with the listeners finding difficulty with segmenting the randomised onset times, and remaining influenced by the acoustic intensity changes, such that a rise in acoustic intensity enhances the likelihood of a tap. We had no directional hypothesis concerning the impact of *Time* (within an excerpt), but consider it feasible that in some circumstances listeners become more confident over time, and hence tended to tap more in a given condition, while in others they become more disinterested (and hence the converse).

Piece 6 comprises randomised prime number time event intervals, in one strand. It had greater event sparsity and less tapping than piece 3. It had no AR, and the standard model gave a Bayesian R^2^ of 0.772. Here, Event_Onsets were influential (estimate = 0.29, evidence ratio = 47.91), as again was tAfterOnset (estimate=-0.11, evidence ratio = 36.82), both evidence ratios are considered strong for a unidirectional hypothesis). The remaining strongly evidenced influence was RMSL1 (estimate = 0.18, evidence ratio = 17999).

We now turn from the dense event set to the sparsely pulsed stimuli set. Piece 11 is part of a rendering of Stockhausen’s Set Sail for the Sun (an improvisatory piece). Computational event onsets were quite sparse here, indeed the longest gap between adjacent pairs was 24 seconds. The standard model (AR2 in this case) gave a Bayesian R^2^ of 0.468, and the only strongly evidenced influences were spectral centroid (estimate = 0.20, evidence ratio=∞, probably best interpreted here as the number of data points, 179, after the removal of the first two of 181, because of time series lagging for the RMS predictor); spectral flatness (estimate=-0.23, evidence ratio = 179), and Time (estimate=-0.04, evidence ratio = 42.24). Other predictors were consistent with hypotheses but not strongly evidenced. The result suggests clearly that segmentation can occur away from Event_Onsets, primarily as a result of transformation of the sound spectrum. Acoustic intensity profiles generally assist, but in this case, not in a strongly evidenced way.

Xenakis’ Bohor (piece 14) was relatively poorly fit, and to avoid autocorrelated residuals it was necessary to use AR1 together with AR5 (the latter coded by forming an additional variable, Tap_Count_L5, at the expense of the loss of the first five data points. The resultant Bayesian R^2^ = 0.305, and the only strongly evidenced predictor was centroid (estimate = 0.08, evidence ratio = 20.09). Spectral flatness showed a negative coefficient as commonly, but the evidence ratio (15.95) was not strong.

The Rag puriya-kalyan (excerpt 24) is the piece with the largest number of large peaks in the tapping counts, as well as being a sparse work, with clear delineation of its harmonic and melodic layers. The standard model (with AR1 and AR6) was successful with a Bayesian R^2^ = 0.856. The model was consistent with expectations, with the following strongly evidenced: Event_Onsets (coefficient = 0.21, evidence ratio = 289.91); RMS (estimate = 0.13, evidence ratio = 1065.67); RMSL2 (estimate=-0.07, evidence ratio = 1141.86; and illustrating the normal alternation of signs of the RMS lag responses); spectral centroid (estimate = 0.36, evidence ratio=∞); spectral flatness (estimate=-0.57, evidence ratio=∞); spectral flux (estimate=-0.11, evidence ratio = 71.07); spectral complexity (estimate = 0.09, evidence ratio = 2284.71); and Time (estimate = 0.11, evidence ratio=∞).

The Sufi meditation (25) was the piece with the second longest mean inter-tap interval (17.34 seconds; Table 4) and had only 9 computed Event_Onsets. The standard model (with AR1) gave a Bayesian R^2^ of 0.626, which is good. Strongly evidenced predictors were: RMSl=L1 (estimate=-0.12, evidence ratio = 1065.67); spectral centroid (estimate = 0.09, evidence ratio = 23.50); spectral flux (estimate = 0.20, evidence ratio=∞); and spectral complexity (estimate = 0.08, evidence ratio = 726.27). The different spectral features thus vary in their relative influence, and with the timbrally varied wind-playing here, the results are comprehensible. Similarly, the temporal profile of the impact of RMS and its lags, varies from piece to piece as well as between people, and hence the oscillation of the positive and negative coefficients amongst the three lags we use as predictors is also familiar and comprehensible.

As might be expected, the spectral components come into their own in terms of statistical likelihood (evidence ratios) particularly in the textural pieces such as 11, 14, 24, 25. Given sparseness, or irregular timings, Event_Onsets may be powerful, but they are also often well captured in RMS profiles, and hence these are very commonly influential over several lags, with a mono- or biphasic time profile, eventually declining.

By multilevel analyses of grouped individual performances and pieces (all data retained bar those excluded as a result of lagging the RMS predictor) in the following section, we will assess further these generalities.

#### Bayesian multilevel time series models of a binary tap indicator, for pieces taken together as two separate groups, dense vs. sparse

We studied separately the two sets of pieces we had established (1–10, 26; and 11–25) representing respectively dense event rhythmic approaches, and more spacious and sparse approaches. Tap occurrence (binary) was modelled with a Bernoulli distribution, and the possible influence of participant liking, familiarity, perceived difficulty, and age was assessed, with a basic formula of the form: Tap ~ Age * Event_Onset + tAfterOnset + RMS + RMSL1 + RMSL2 + Centroid + Flatness + Flux + Spectral_Complexity + Liking + Familiarity + Difficulty + Index + AR(*p* = 4) + (1 | Participant) + (1 | Track_ID). This was derived from preliminary selective modelling, as providing acceptably good overall fit (eventual Bayesian R^2^ for both models was 0.61).

For the dense group (Table 7), we found no strong evidence for the positive effects of onsets (consistent with comments above that in items 1 and 26, these are so frequent and recurrent that they probably do not create strong segmentation points, while in the remaining stimuli they are too irregular to be strongly predicted and hence perhaps create segment closure). Spectral flux, spectral complexity, and Time were strongly evidenced positive predictors; and negative effects of tAfterOnset were again observed. These results were resoundingly consistent with the observations above. The participant responses for Age, Liking, Familiarity, and Difficulty were not strongly evidenced, while the Age: Onset (rhythm_marker) interaction was (but with only a relatively small coefficient of 0.09).

Table 7 and 8. Standard models of tapping to the rhythmic (Table 7) and the spacious (Table 8) group of pieces. For both, AR = 4 (autoregression order 4). The same R formula was used for both models.

**Table 7:** Dense events (items 1–10, 26). Hypothesis tests only for the strongly evidenced predictors. An AR4 structure was effective. The Bayesian R^2^ = 0.61

Family: Poisson Links: mu = log Formula: tap_count ~ Age*Event_Onsets + tAfterOnset + RMS + RMSL1 + RMSL2 + Centroid + Flatness + Flux + Spectral_Complexity + Index + Liking + Familiarity + Difficulty + AR(*p* = 4) + (1 | PID) + (1 | Track_ID)							
Hypothesis	Estimate	Est. Error	CI. Lower	CI. Upper	Evid. Ratio	Post.Prob	Star
Age * Event_Onsets > 0	0.09	0.04	0.04	0.1	13999.00	1.00	*
tAfterOnset < 0	-0.49	0.16	-0.80	-0.30	Inf	1.00	*
Flux > 0	0.40	0.15	0.20	0.68	Inf	1.00	*
Spectral_Complexity > 0	0.39	0.18	0.18	0.71	799.00	1.00	*
Index > 0	0.42	0.18	0.18	0.74	1646.06	1.00	*

**Table 8:** Sparse events (items 11–25). Hypothesis tests only for the strongly evidenced predictors. Again, an AR4 structure was effective. The Bayesian R^2^ = 0.61

Hypothesis	Estimate	Est. Error	CI. Lower	CI. Upper	Evid. Ratio	Post. Prob	Star
Event_Onsets > 0	0.53	0.30	0.03	1.02	23.10	0.96	*
Age * Event_Onsets > 0	0.02	0.01	0.00	0.05	32.85	0.97	*
tAfterOnset < 0	-0.02	0.01	-0.04	-0.01	23999.00	1.00	*
RMS + RMSL1 + RMSL2 > 0	0.46	0.11	0.29	0.64	Inf	1.00	*
Centroid > 0	1.56	0.14	1.33	1.80	Inf	1.00	*
Flatness < 0	-1.77	0.14	-2.01	-1.54	Inf	1.00	*
Flux > 0	0.31	0.10	0.14	0.47	959.00	1.00	*
Spectral_Complexity > 0	0.22	0.08	0.10	0.35	614.38	1.00	*
Index > 0	0.37	0.05	0.29	0.46	Inf	1.00	*

The sparse set (Table 8) also shows results consistent with earlier observations on them. For these excerpts, Event_Onsets themselves are positive predictors of segmentation, while tAfterOnset shows a strongly evidenced but negligible negative coefficient (estimate=-0.02, evidence ratio = 23999.00), both features contrasting strongly with those of the dense group. Interestingly, RMS and Centroid became positive predictors in the sparse event model, joining Flux and Spectral_Complexity. Spectral Flatness also emerged as negative predictor in the sparse model, although there was no strong evidence of an effect of Flatness on the dense group. The participant features/evaluations like Age, Liking, Familiarity, and Difficulty were again non-influential, and the interaction terms between Age*Event_Onset, while again strongly evidenced, was again slight (estimate = 0.02).

When we considered the two groups together, we found very strong evidence of a positive influence of Event_Onsets on tap count for the sparse group. Conversely, we found no evidence that Event_Onsets influenced tap count for the dense group. This may conceivably be explained by the fact that for some dense excerpts, Event_Onsets have occurred in virtually every 500ms time slice, reducing the sensitivity of the model to detect onset-driven effects, rather than implying no perceptual effect. However, Event_Onsets may well be perceptually somewhat irrelevant to segmentation in densely music as listeners may use segmentation strategies from other acoustic/perceptual cues, such as intensity or timbre changes, and other acoustic features that are less susceptible to having their variability reduced when down sampled, as we assess.

Consistent with this, the negative tAfterOnset decay of tapping likelihood is much smaller for the sparse excerpts (operating generally over a longer time range). Also consistent with this, AR1 of both groups is also negative, while the sum of AR1-4 is positive. The overall positive effect of RMS lags, Centroid, Flux, Spectral_Complexity and Time are also increased in the sparse group, as is the negative impact of spectral Flatness. In sum, in the sparse group of pieces, segmentation is more substantially influenced by the progressions of the acoustic intensity and the spectral features, than in the dense events rhythmic group, even though the acoustic features have been standardised for these model analyses, so that differences in their distributions have been to some degree reduced. In many of the sparse pieces, there is more timbral contrast than in the dense group pieces.

Given prior suggestions (Carson, 2007) that people may imagine regular rhythmic structure even when listening to music that lacks it (e.g. randomised note attack times), we also wanted to assess whether in the sparse group of pieces there is any indication of such regularity in perceived segment durations. The high CVs (Table 4) of the inter-tap intervals across participants in both the rhythmically complex and most of the sparse pieces already suggest that temporal regularity declines in these circumstances. One strong criterion of any tendency to tapping regularity would be an assessment of whether tapped segments are more consistent in duration than Event_Onsets (note that this is not to imply closeness of taps to onsets). This possible consistency can be judged by assessing the coefficient of variation (CV) of the two features (that is their IOI s.d./mean): if the tapped segments show lower CV than the corresponding onsets, this might be a positive indicator of such a tendency, and vice versa. So, response by response we measured the two CVs and compared them (unlike the preceding analyses, this used all the tap and onset data, keeping all performances separate in the data, and there was no time slicing (so Vamp-detected pairs of taps close in time would be accounted for). The 65 participants and 15 pieces in the sparse pulse group showed only 4 performances (out of 954 that could be analysed) where the tapping IOI CV was lower than the Event_Onset IOI CV. The range of the Event_Onset IOI CVs (of course, one observation per piece) was 0.006–0.131. The range of the average tapping CVs, where each piece gained a performance from every one of the 65 participants (bar 21 lost or failed performances) was 0.616–0.832. So overall there was negligible evidence implying a tendency of participants to superimpose rhythmic regularity on their perceptions of segment length in the sparse (and unpulsed) extracts. Finally, the GMSI scores were not predictive in models.

## Discussion

Our data were clear that musical excerpts characterised by different event-densities, and/or by different pulsedness, were segmented very differently by our participants, with more variability between participants in the event-sparse examples. Audible event onsets clearly encouraged the decision to tap to represent the conclusion of a segment, but not surprisingly, when very dense, for example such that there was an event in every time slice, this became relatively unimportant, while other acoustic nuances supported the decisions to recognise the completion of a segment. Conversely, in the event-sparse music, individual events were more influential, and there was a clear decline in likelihood to define a segment end with time after an event. Acoustic features associated with commencement of events or their density, such as acoustic intensity over a time slice, were positive influences. Features of spectral brightness and variability (Complexity and Flux) were positive predictors, while spectral noise content (Spectral Flatness) was negative. Overall, different listening strategies between the two sets of music (sparse vs. dense) were implied.

IOIs of tapping were large in each group of stimuli, particularly in the sparse group, but in both cases were highly variable between participants (s.d. >> mean; Table 3) indicating that even for the dense set, some of which at least were clearly metrical, segmentation of the piece need not necessarily be driven by metrical structure (i.e. beats). As rhythmic complexity increases (by introducing randomised timings), we found that tap density reduced, suggesting a propensity to focus on density and IOI (Milne et al., 2021), which can usefully lead to perceived structural simplification (Møller et al., 2021). Simplification is probably guided by several factors: expertise (Zhang et al., 2016), prior exposure (Ayari & McAdams, 2003; Lartillot & Ayari, 2011; Popescu et al., 2021), and the acoustic content of the piece (Landy, 2011). Tapping to reproduce a rhythm is thus, unsurprisingly, not a direct approach to assessing either rhythmic or overall musical segmentation by listeners, though it may provide data components relevant to both.

Our experiment was not designed to test the influence of expertise or experience, but given the data gathered it was nevertheless critically modelled in this respect. For example, one might expect that a higher degree of familiarity with a piece or style would allow a listener to use more complex strategies of segmentation, in our case expected to enhance tapping rates, as was previously found in the tapping of more common duple and triple meters (Milne et al., 2021). Thus, while we expected to reveal a positive interaction between familiarity and the effect size associated with events, this proved to not be the case: our findings showed no evidence that personal features, such as age, liking, familiarity, and perceived difficulty influenced tapping rates, or were indicative of changes in segmentation strategy for either the sparse or dense groups. This may stem from the fact that our stimuli were rather diverse and may have elicited comparably diverse personal ratings, overshadowing any potential influence on tapping behaviour.

Overall, our findings indicate that the underlying mechanisms governing segmentation involve a complex interplay between the density and timing of audible events, and the acoustic cues used to parse both sparse and dense music. It appears that segmentation becomes harder at the extreme ends of the sparse/dense continuum: for extremely sparse pieces there may well be a paucity of audible events and/or metrical, timing, and acoustic cues, while for extremely dense pieces there may be an excess of all of these. In some senses it seems that segmentation strategies for the sparse pieces rely upon complication, while for the dense, they rely on simplification. Whether there is a segmentation ‘sweet spot’ in the middle of the continuum may be a matter of individual taste.

## Supplementary Information

Below is the link to the electronic supplementary material.

Supplementary Material 1 (PDF 134 KB)

## Data Availability

The datasets generated during and/or analysed during the current study, together with pre-experiment questionnaires, audio and visual stimuli are available in the Open Science Framework repository, https://osf.io/ac4gu.

## Declarations

### Reprints and permission information

is available at http://www.nature.com/reprints.
