# Supplementary material for: Comparing perceptual segmentation in pulsed and unpulsed music with diverse event densities
Source: Psychol Res. 2026 Sep 11;90(5):165. doi: 10.1007/s00426-026-02371-w (PMC13569503; doi:10.1007/s00426-026-02371-w)
Supplement: Supplementary file 1 [file 426_2026_2371_MOESM1_ESM.pdf]

Supplementary

Supplementary S1. Pre-Experiment Survey

Q1. What is your age?

____

Q2. What is your gender?

- Female (1)
- Male (2)

Q3. What is your Nationality?

____

Q4. Country in which you spent the formative years of your childhood and youth:

____

Q5. Country of current residency:

____

Q6. What is the Highest educational qualification you have attained? (Please select one)

- Did not complete any school qualification (1)
- Completed first school qualification at about 16 years (e.g. GCSE/Junior High School) (2)
- Completed Second qualification (e.g. A levels/ High School) (3)
- Undergraduate degree or professional qualification (4)
- Postgraduate degree (5)
- I am still in education (6)

Q7. If you are still in education, what is the highest qualification you expect to obtain? (Please select one)

- First school qualification (e.g. GCSE / Junior High School) (1)
- Post-16 vocational course (2)
- Second school qualification (e.g. A-levels / High School) (3)
- Undergraduate degree or professional qualification (4)
- Postgraduate degree (5)
- NA - I am not in education (6)

Q8. Please select the most appropriate category:							
	Completely Disagree (1)	Strongly Disagree (2)	Disagree (3)	Neither Agree nor Disagree (4)	Agree (5)	Strongly Agree (6)	Completely Agree (7)
I spend a lot of my free time doing music-related activities.							
I sometimes choose music that can trigger shivers down my spine.							
I enjoy writing about music, for example on blogs and forums.							
If somebody starts singing a song I don't know, I can usually join in.							
I am able to judge whether someone is a good singer or not.							
I usually know when I'm hearing a song for the first time.							
I can sing or play music from memory.							
I'm intrigued by musical styles I'm not familiar with and want to find out more.							
Pieces of music rarely evoke emotions for me.							
I am able to hit the right notes when I sing along with a recording.							

Q9. Please select the most appropriate category:							
	Completely Disagree (1)	Strongly Disagree (2)	Disagree (3)	Neither Agree nor Disagree (4)	Agree (5)	Strongly Agree (6)	Completely Agree (7)
I find it difficult to spot mistakes in a performance of a song even if I know the tune.							
I can compare and discuss differences between two performances or versions of the same piece of music.							
I have trouble recognizing a familiar song when played in a							

different way or by a different performer.							
I have never been complimented for my talents as a musical performer.							
I often read or search the internet for things related to music.							
I often pick certain music to motivate or excite me.							
I am not able to sing in harmony when somebody is singing a familiar tune.							
I can tell when people sing or play out of time with the beat.							
I am able to identify what is special about a given musical piece.							
I am able to talk about the emotions that a piece of music evokes for me.							

Q10. Please select the most appropriate category:							
	Completely Disagree (1)	Strongly Disagree (2)	Disagree (3)	Neither Agree nor Disagree (4)	Agree (5)	Strongly Agree (6)	Completely Agree (7)
I don't spend much of my disposable income on music.							
I can tell when people sing or play out of tune.							
When I sing, I have no idea whether I'm in tune or not.							
Music is kind of an addiction for me - I couldn't live without it.							
I don't like singing in public because I'm afraid that I would sing wrong notes.							
When I hear a piece of music, I can usually identify its genre.							
I would not consider myself a musician.							
I keep track of new music that I come across (e.g. new artists or recordings).							
After hearing a new song two or three times, I can usually sing it by myself.							

I only need to hear a new tune once and I can sing it back hours later.							
Music can evoke my memories of past people and places.							

Q11. I engaged in regular, daily practice of a musical instrument (including voice) for how many years? (Please select one)

- 0 years (1)
- 1 year (2)
- 2 years (3)
- 3 years (4)
- 4-5 years (5)
- 6-9 years (6)
- 10 or more years (7)

Q12. At the peak of my interest, I practiced for how many hours per day on my primary instrument? (Please select one)

- 0 hours (1)
- 0.5 hours (2)
- 1 hour (3)
- 1.5 hours (4)
- 2 hours (5)
- 3-4 hours (6)
- 5 or more hours (7)

Q13. I have attended how many live music events as an audience member in the past twelve months. (Please select one)

- 0 (1)
- 1 (2)
- 2 (3)
- 3 (4)
- 4-6 (5)
- 7-10 (6)
- 11 or more (7)

Q14. I have had formal training in music theory for how many years. (Please select one)

- 0 years (1)
- 0.5 years (2)
- 1 year (3)
- 2 years (4)
- 3 years (5)
- 4-6 years (6)
- 7 or more years (7)

Q15. I have had how many years of formal training on a musical instrument (including voice) during my lifetime. (Please select one)

- 0 years (1)
- 0.5 years (2)
- 1 year (3)
- 3-5 years (4)
- 6-9 years (5)
- 10 or more years (6)

Q16. I can play how many musical instruments. (Please select one)

- 0 (1)
- 1 (2)
- 2 (3)
- 3 (4)
- 4 (5)
- 5 (6)
- 6 or more (7)

Q17. I listen attentively to music for how long per day. (Please select one)

- 0-15 minutes (1)
- 15-30 minutes (2)
- 30-60 minutes (3)
- 60-90 minutes (4)
- 2 hours (5)
- 2-3 hours (6)
- 4 hours or more (7)

Q18. The instrument I play best (including voice) is

____

Q19. Please tick one of the following: (occupational status)

- Still at School (1)
- At University (2)
- In Full-time employment (3)
- In Part-time employment (4)
- Self-employed (5)
- Homemaker/full time parent (6)
- Unemployed (7)
- Retired (8)

Q20. What is the musical genre you mainly listen to? (Please select one)

- Rock/Pop (1)
- Jazz (2)
- Classical Music (3)
